# Breastfeeding performance in Afar regional state, northeastern Ethiopia: a cross sectional study

**DOI:** 10.1186/s12887-018-1353-y

**Published:** 2018-11-30

**Authors:** Jemal Hussien, Selam Assefa, Misgan Legesse Liben

**Affiliations:** 1 Health and Nutrition specialist department, Save the children, Afar, Ethiopia; 2 School of Public Health, College of Health Sciences, Mekelle University, Tigray, Ethiopia; 3 Department of Public Health, Faculty of Health Sciences, Woldia University, Amhara, Ethiopia

**Keywords:** Breastfeeding performance index, Exclusive breastfeeding, Semera-logia city, Afar, Ethiopia

## Abstract

**Background:**

In Ethiopia fewer than 20% of infants aged less than six months are appropriately fed. This study aimed to identify predictors of poor breastfeeding performance among mother-infant pairs in Samara-Logia city administration, Afar Regional State, Ethiopia.

**Methods:**

Five hundred and seventy six mothers of infants aged less than six months were recruited to participate in face-to-face structured interviews at their home. Infant feeding practices were measured by breastfeeding performance index (BPI). Infants who scored 0–3 BPI were classified as achieving ‘Low BPI’, 4–5 as ‘Medium BPI’, and 6–7 scores as ‘High BPI’. A pre-tested instrument was used to assess demographic characteristics of mothers and their experiences of infant feeding. EpiData version 3.02 was used to enter, clean and code the data. Then, data were analyzed using SPSS version 20.

**Results:**

Five hundred thirty six (93.1%) of the study mothers had ever breastfed their infants. About 83% [95% CI: 80.0, 86.0%] of infants had achieved low or medium BPI scores. Being older than 34 years [AOR: 4.55; 95% CI (1.33, 15.73)], having an infant aged 4–5 months [AOR: 2.49; 95% CI (1.08, 5.76)], giving birth at home [AOR: 4.25; 95% CI (1.37, 13.23)] or by caesarean section [AOR: 8.00; 95% CI (2.78, 23.09)] and receiving advice on proper infant feeding practices at postnatal checkup [AOR:0.28; 95% CI (0.13,0.59)] were independent predictors of low/medium BPI scores.

**Conclusion:**

This study revealed that nearly eight in ten infants aged less than six months achieved low/medium BPI scores. Being older than 34 years, having an infant aged 4–5 months, giving birth at home or by caesarean section and lack of advice on proper infant feeding practices were associated with higher odds of low/medium BPI scores.

## Background

Human milk provides the necessary nutrients for optimal growth and development in the first six months; hence breastfeeding is the ultimate infant feeding approach and has important infant and maternal health advantages [1, 2]. For instance, breastfeeding has well-established short-term benefits, particularly in the reduction of morbidity and mortality due to infectious diseases in childhood. Breastfeeding protects against diarrhea incidence and respiratory infection [3].

Globally, about 35% deaths among under-five children are attributed to under nutrition. More than two third of neonatal deaths are attributed to inappropriate infant feeding practices [4]. Low and medium BPI scores are responsible for 45% of infectious, 30% of diarrheal and 18% of acute respiratory deaths among preschool children [4, 5]. A meta-analysis showed association between breastfeeding and minimum prevalence of overweight later in life. In addition, breastfeeding have a protective effect against diabetes among adolescents [6].

Exclusive breastfeeding is an infant’s consumption of only breast milk but not any type of foods or drinks, except for medical indications in the first 6 months [7]. Compared to infants who were exclusively breastfed, infants who were not exclusively breastfed have 88% higher risk of mortality. Breastfeeding protects infant from hospital admission [8]. Moreover, above 13% of mortality among preschool children can be prevented by providing exclusive breastfeeding [7].

Breastfeeding performance index (BPI) is measured by seven measurements; early breastfeeding initiation, prelacteal feeding, current breastfeeding, bottle feeding, and any liquid (except medicine), any solid food and formula given in the last 24 h [9].

Ethiopia has adapted infant and young child feeding guideline since 2004 [10], and the national nutrition program in 2013 [11] to unlock the lifesaving potential of optimal breastfeeding practices through multi-sectoral approaches. However, fewer than 20% of infants in Ethiopia are appropriately fed during the first six months of life, and lower BPI was associated with increased risks of diarrhea and fever [12]. This study aimed to identify predictors of poor breastfeeding performance among mother-infant pairs in Samara-Logia city administration, Afar Regional State, Ethiopia.

## Methods

### Study setting and period

A community based cross sectional study was conducted from February 11–25/2017 in Semera-Logia city administration of Afar Regional State. Semera-Logia city administration is located at 574 k meters from Addis Ababa (the capital of Ethiopia). The city administration has thirteen ketenas (the smallest administrative units next to kebele in Ethiopia).

According to the national statistics population projection for 2016/2017, the city administration has a total population of 45,980 in which 21,610 were females. Based on Afar Regional Health Bureau estimate, in the city administration the number of the target mother-infant pairs was estimated to be 1380. There are 2 health centers and 13 private clinics in the city administration.

### Sample size determination and sampling procedure

A sample size of 610 was determined using the following formula;

$$ \mathrm{n}=\mathrm{D}\left[\frac{{\left(\mathrm{z}\frac{\upalpha}{2}\right)}^2\mathrm{p}\left(1-\mathrm{p}\right)}{{\mathrm{d}}^2}\right] $$

**Assumptions:** n = required sample size, $\mathrm{z}\frac{\upalpha}{2}$ = critical value for normal distribution at 95% confidence level (1.96), P = prevalence of low/medium BPI score in Ethiopia (76.4%) [12], d = 0.05 (margin of error), D = 2 (cluster effect) and 10% for nonresponse.

In this study a ketena was defined as a cluster. Then, the city administration was divided in to thirteen clusters. Then, six clusters were randomly selected by lottery method. All mothers of infants aged less than six months in the selected clusters were included. Women who were unable to communicate and non-biological were not included in the study.

### Study variables

Breastfeeding performance was the outcome variable measured by breastfeeding performance index (BPI). BPI scores were constructed by allocating one point for each of the following: early breastfeeding initiation; complete avoidance of prelacteal feeds; complete avoidance of feeding bottles; current breastfeeding; not receiving liquids; not receiving formula or other milk; and not receiving solids in the last 24 h [9]. Scores for each item on the BPI were summed to give a total score that could range between 0 and 7). Infants who scored 0–3 BPI were classified as achieving ‘Low BPI’, 4–5 as ‘Medium BPI’, and 6–7 scores as ‘High BPI’ [12].

Independent variables: maternal age, child age, parity and gravidity were continuous variables. Categorical variables were: maternal related variables (religion, ethnicity, occupation, educational and marital status), antenatal care (ANC) attendance, postnatal care (PNC) attendance, infant feeding counseling services at PNC or ANC checkups, paternal educational status, child’s sex, household head, site and mode of delivery.

### Data collection tools and procedures

Data were collected using structured and interviewer administered questionnaire adapted from the EDHS [13] and other related literatures [12, 14–23]. It was prepared first in English then translated in to Afar’af. The data collectors (10 diploma nurses) were trained for two days on the questionnaire, consent procedures and interview techniques. The questionnaire was pretested on one ketena, which was not included in the study. Then, the questionnaire was improved and contextualized to fit the local condition and the study objective. Finally, Afar’af version of the questionnaire was used to collect the data.

### Data management and processing

Data were checked for completeness and inconsistencies. EpiData version 3.02 was used to enter, clean and code the data. Then, SPSS version 20 was used to analyse the data.

The crude odds ratio (COR) was estimated in the simple logistic regression analysis. Variables with *p*-value < 0.3 in the simple logistic regression analysis were included in the multivariable logistic regression analysis. The Hosmer-Lemeshow goodness-of-fit with backward LR was used to assess the necessary assumptions for the application of multivariable logistic regression analysis, and a good fit test will yield a *p*-value> 0.05*.*

Adjusted Odds Ratio (AOR) with 95% confidence interval was estimated to assess the strength of the association. A *p*-value < 0.05 in the multivariable logistic regression analysis was used declare independent predictors of low/medium BPI score.

### Ethical considerations

This study was approved by the Research Ethics Review Committee (RERC) of Samara University dated 23 Dec 2016, and numbered ERC/0025/2016. An official letter was written from Samara University to the Semera-Logia city administration office. Then, support letters were sent to each selected ketena. A signed consent was taken from the study participants after informing the study subjects on study objectives, expected outcomes, benefits and the risks associated with it. In the case of participants under the age of 18 years, parental/legal guardian consent was taken on the participants’ behalf. Confidentiality of responses was maintained throughout the study.

## Results

### Socio-demographic characteristics of the study subjects

Totally 576 mother-infant pairs participated in the study (estimated response rate 94.4%). The majority of the mothers were Muslims (80.2%) followed by Ethiopian Orthodox Christians (15.3%). More than half of the mothers were aged 20–34 years with mean (+SD) age of 31.79 (±7.18) years. In addition, the mean (±SD) age of the study infants was 3.6 (±1.4) months. About 72% of the households were led by males (Table 1).

**Table 1:** Socio-demographic characteristics of mothers of infants aged less than six months (*N* = 576) in Samara-Logia town Administration, Northeast Ethiopia, 2017

Characteristics	Frequency	Percent
Maternal age (years)		
17–20	35	6.1
20–34	322	55.9
> 34	219	38.0
Maternal religion		
Muslim	462	80.2
Ethiopian Orthodox	88	15.3
Protestant	26	4.5
Ethnicity of the mother		
Afar	133	23.1
Amhara	302	52.4
Oromo	62	10.8
Tigray	79	13.7`
Marital status		
Married	456	79.2
Divorced	76	13.2
Widowed	31	5.4
Single	13	2.2
Maternal Education		
None	128	22.2
Primary	175	30.4
Secondary and above	273	47.4
Maternal Occupation		
Housewife	242	42
Government employee	220	38.2
Merchant	64	11.1
Daily laborer	36	6.3
Others^a^	14	2.4
Age of the infant (months)		
0–1	53	9.2
2–3	227	39.4
4–5	296	51.4
Birth order		
1	132	22.9
2	247	42.9
> 3	197	34.2
Paternal education		
None	54	11.8
Primary	52	11.4
Secondary and above	350	76.8
Household head		
Mother of index infant	159	27.6
Father of index infant	417	72.4
Having Radio		
Yes	199	34.5
No	377	65.5

^a^Student, agro-pastoralist. *ETB* Ethiopian Birr

### Maternal health service utilization

Three hundred and seventy five (65.1%) of mothers had attended at least one ANC check-up, with only 7.5% attending four or more visits. About 40% of the study infants were delivered at home (Table 2).

**Table 2:** Maternal health service utilization and obstetric history among mothers of infants aged less than six month, in Samara-Logia town, Northeast Ethiopia, 2017

Variables	Frequency	Percent
ANC checkup^a^		
Yes	375	65.1
No	201	34.9
Number of ANC checkups		
1	202	53.8
2	102	27.2
3	43	11.5
> 4	28	7.5
Mothers counseled on infant feeding at ANC checkup		
Yes	319	85.1
No	56	14.9
Place of delivery		
Home	229	39.8
Health institution	347	60.2
Mode of delivery		
Vaginal	521	90.5
Cesarean section	55	9.5
Parity		
Primipara	178	30.9
Multipara	398	69.1
PNC checkup^a^		
Yes	264	45.8
No	312	54.2
Mothers counseled on infant feeding at PNC checkup		
Yes	245	92.8
No	19	7.2

^a^at least one checkup. *ANC* Antenatal care. *PNC* postnatal care

### Knowledge and awareness on infant feeding practices

Five hundred sixty one (97.4%) of the study participants had heard about recommended infant feeding practices. Nearly 90% of the mothers reported that breastfeeding has benefits for their infants. However, 45.3% of mothers reported that it is good to provide feeds other than breast milk immediately at birth (Table 3).

**Table 3:** Knowledge and awareness of mothers of infants aged less six months on infant feeding practices (*N* = 576) in Samara-Logia town, North East Ethiopia, 2017

Variables	Frequency	Percent
Heard About infant feeding recommendations		
Yes	561	97.4
No	15	2.6
Breastfeeding benefits for the health of infants		
Yes	515	89.4
No	61	10.6
Breastfeeding benefits for the health of the mother		
Yes	333	57.8
No	243	42.2
Newborn should start breastfeeding within 1 h after birth		
Yes	356	61.8
No	220	38.2
Colostrum should be given to the newborn		
Yes	377	65.5
No	199	34.5
Providing feeds other than breast milk at birth is good to the newborn		
Yes	261	45.3
No	315	54.7
Mothers breast milk is enough without addition of water and/or other fluids in the first six months		
Yes	335	58.2
No	241	41.8
The infant should start complementary food at six completed months		
Yes	346	60.1
No	230	39.9

### Infant feeding practices and breastfeeding performance index (BPI) score

Five hundred thirty six (93.1%) of the study mothers had ever breastfed their index infants. About 45% of the study mothers had initiated breastfeeding within an hour after birth. More than half (62.5%) of the infants had received prelacteal feeds within three days after birth, while 66.8% of infants fed on a bottle with a nipple. In addition, 45.7 and 37.2% of infants were introduced to fluids and solid foods before six months of age, respectively.

The mean (+SD) of BPI score was 3.41(+ 0.69). More than half (56.8%) of mothers achieved low BPI scores, around a quarter (25.8%) achieved medium scores, and fewer than one in five achieved high BPI scores (Table 4).

**Table 4:** Feeding practices of mothers of infants aged less than six months and BPI score (*N* = 576) in Samara-Logia town, Northeast Ethiopia, 2017

Variables	Frequency	Percent	Score
Ever breastfeeding			
Yes	536	93.1	
No	40	6.9	
Early breastfeeding initiation			
No	316	54.9	0
Yes	260	45.1	1
Current breastfeeding			
No	256	44.4	0
Yes	320	55.6	1
Prelacteal feeding			
Yes	360	62.5	0
No	216	37.5	1
Bottle feeding			
Yes	385	66.8	0
No	191	33.2	1
Liquids given			
Yes	263	45.7	0
No	313	54.3	1
Formula milk given			
Yes	271	47.0	0
No	305	53.0	1
Solid foods given			
Yes	214	37.2	0
No	362	62.8	1
Over all BPI score			
Low	327	56.8	
Medium	149	25.8	
High	100	17.4	

*BPI* Breastfeeding Performance Index

### Factors associated with low/medium BPI score

Mothers aged older than 34 years were about four times more likely to achieve low/medium BPI score as compared to mothers younger than 20 years. Mothers of infants aged 4–5 months were 2.5 times more likely to achieve low/medium BPI score as compared to those mothers having infants under 2 months. Women who gave birth at home or by cesarean section had more odds of low/medium BPI score achievement as compared to their counter parts. Mothers who received infant feeding advice at postnatal checkup were 77% less likely to achieve low/medium BPI score (Table 5).

**Table 5:** Factors associated with low/medium BPI scores among mothers of infants aged less than six months in Samara-Logia town administration, Afar Regional state, Northeast Ethiopia, 2017

Variables	Low/medium BPI score		COR (95% CI)	AOR (95% CI)
	Yes	No		
Maternal age (years)				
17–20	29	6	1	1
20–34	248	74	0.69(0.28,1.73)	1.06(0.34,3.28)
> 34	199	20	2.06(0.76, 5.55)	4.55(1.33, 15.73)*
Maternal occupation				
Housewife	193	49	0.71(0.46,1.09)	
Other^a^	283	51	1	
Maternal educational status				
Non formal education	118	10	2.97(1.49,5.89)*	
Formal education	358	90	1	
Religion				
Muslim	378	84	0.74(0.41,1.31)	
Christian^b^	98	16	1	
Household head				
Mother of index infant	142	17	2.08(1.19,3.63)*	
Father of index infant	334	83	1	
Ethnicity				
Amhara	240	62	1	
Afar	112	21	1.38(0.80, 2.37)	
Oromo	53	9	1.52(0.71, 3.25)	
Tigre	71	8	2.29(1.05, 5.01)*	
Parity				
Primipara	167	11	1	
Multipara	309	89	0.23(0.12,0.44)*	
Marital status				
Currently married	373	83	1	
Currently unmarried	103	17	1.35(0.77,2.37)	
Age of infant (month)				
0–1	35	18	1	1
2–3	188	39	2.48(1.28,4.82)*	2.18(0.95,5.00)
4–5	253	43	3.03(1.57,5.82)*	2.49(1.08,5.76)*
Birth order				
1	117	15	1	
2	194	53	0.47(0.25,0.87)*	
> 3	165	32	0.66(0.34,1.28)	
Advice on infant feeding at ANC checkup				
Yes	232	87	0.14(0.08, 0.26)*	
No	244	13	1	
Place of delivery				
Home	223	6	13.81(5.93,32.14)*	4.25(1.37,13.23)*
Health institution	253	94	1	1
Mode of delivery				
Vaginal	426	95	1	1
Cesarean section	50	5	2.23(0.87,5.74)	8.00(2.78,23.09)*
Advice on infant feeding at PNC checkup				
Yes	167	78	0.15(0.09, 0.25)*	0.28(0.13,0.59)*
No	309	22	1	1

Hosmer and Lemeshow Test = 0.455. COR = Crude odds ratio. *AOR* Adjusted odds ratio. *CI* confidence interval. *Significant at *p* < 0.05. ^a^student, agro-pastoralist, merchant, government employee, daily laborer. ^b^Ethiopian Orthodox, Protestant. *ANC* Antenatal care. *PNC* postnatal care

## Discussion

More than half (56.8%) of mothers achieved low BPI scores, around a quarter (25.8%) achieved medium scores, and fewer than one in five achieved high BPI scores. This is relatively similar with a finding in Ethiopia, where 80% of infants achieved low/medium BPI score [12]. This can be justified by high prevalence of infant feeding malpractices in the country. For instance, prelacteal feeding and colostrum discarding are common in different cultures of Ethiopia. In Afar Regional State, nearly 43% of children aged 6–23 months received prelacteal feeding [22]. In northeastern Ethiopia, colostrum is assumed to cause abdominal cramp; therefore, grandmothers and traditional birth attendants influence mothers to discard colostrum, and to nourish infants with prelacteal feeds [24].

This study showed that as infants got older the overall breastfeeding performance index (BPI) score decreases. Mothers of infants aged 4–5 months were 2.5 times more likely to achieve low/medium BPI score as compared to those mothers having infants aged less than 2 months. Similar findings were also reported at Cameroon [16], Dubti town [17] and Hawassa of Ethiopia [18]. It is not surprising that children aged 4–5 months were more likely to achieve low/medium BPI score. They are on the cusp of transition to complementary foods and many are likely to have received formula and solid foods as mothers may perceive them to be ready for complementary foods. In addition, traditional postpartum care is given in the first few months of delivery where women stay at home with their infants; providing the chance of proper breastfeeding that will increase the BPI score.

Mothers aged older than 34 years were about five times more likely to achieve low/medium BPI score as compared to mothers younger than 20 years. In North Wollo zone of Ethiopia, eldest mothers were more likely to practice infant feeding malpractices as compared to youngest mothers [25, 26]. This can be justified in such a way that first time mothers learn about child care from older female family members [27]. However, grandmothers and traditional birth attendants recommend colostrum avoidance and prelacteal feeding [24, 27]. This supports the finding that older females are more likely to achieve low/medium BPI score.

Compared to women who gave birth at health institution, women who delivered at home were about four times more likely to achieve low/medium BPI score. Likewise, in Amhara region of Ethiopia, higher odds of early initiation of breastfeeding were noted among mothers who gave birth at a health institution compared to those who did it at home [19, 20]. This might be due to the fact that home delivery provides favorable environment for family and community influence on mothers to practice infant feeding malpractices. Therefore, it is important to educate traditional birth attendants and/or households members who exert influence on mothers’ behaviors [24].

Women who delivered the study infants by cesarean section had more odds of achieving low/medium BPI score as compared to their counter parts. Similar findings were reported at Egypt where caesarean section delivery was an independent predictor of low BPI [4]. In the Amibara district of Ethiopia, caesarean section delivery was positively associated with late initiation of breastfeeding. This can be associated with long recovery time taken by mothers due to anesthesia effects, and infants born by cesarean section might be taken to intensive care unit [21].

Mothers who received advice about infant and young child feeding at postnatal checkup were less likely to achieve low/medium BPI score. In Dubti town, mothers who received counseling during postnatal checkup was associated with increased odds of exclusive breastfeeding [17]. In Afar regional state, lack of awareness on proper breastfeeding practices were associated with increased odds of prelacteal feeding [22]. Likewise, mothers who received postnatal care were more likely to practice exclusive breastfeeding [23]. Furthermore, mothers having high knowledge on infant and young child feeding (IYCF) had more odds of timely breastfeeding initiation compared to mothers having poor IYCF knowledge [20]. These findings highlight the importance and value of providing mothers with infant feeding advice particularly at postnatal care visits.

### Limitations

This study could be subjected to social desirability bias where study participants might tend to report the desired infant feeding recommendations which were not their actual practices. Second, since the data were collected based on self-report of the mothers of infants aged less than six months, it may be subjected to recall bias.

## Conclusions

This study revealed that roughly 8 in every 10 infants achieved low/medium BPI score. Being older than 34 years, having an infant aged 4–5 months, giving birth at home or by caesarean section and lack of advice on proper infant feeding practices were associated with low/medium BPI score. This indicates promoting institutional delivery, supporting breastfeeding education and counseling programs are vital. Hence, health care professionals at antenatal, postnatal and delivery care units should provide counseling on proper infant feeding practices. In addition, appropriate supervision and assistance to health care staffs during cesarean section are important interventions to improve BPI score. Further research is also required to identify and address barriers to institutional delivery and postnatal care service uptake.

## Abbreviations

AOR: Adjusted odds ratio
BPI: Breastfeeding performance index
CI: Confidence interval
COR: Crude odds ratio
ERC: Ethical Review Committee
IYCF: Infant and young child feeding
RERC: Research Ethics Review Committee
